# Concurrent Primary Cardiac Tumors in a High-Risk Patient Presenting With Tamponade

**DOI:** 10.7759/cureus.17324

**Published:** 2021-08-20

**Authors:** Jay V Gopal, Lauren Crowley, Shawn M Quinn, Timothy S Misselbeck, Joseph B Zackary

**Affiliations:** 1 Department of Emergency and Hospital Medicine, Lehigh Valley Health Network, University of South Florida Morsani College of Medicine, Bethlehem, USA; 2 Department of Surgery, Lehigh Valley Health Network, University of South Florida Morsani College of Medicine, Bethlehem, USA

**Keywords:** primary cardiac tumors, atrial myxoma, tamponade, emergency echocardiography, imminent mitral annular obstruction

## Abstract

Primary cardiac tumors are rare, particularly in the elderly population. The patient described in this report presented with symptoms of dyspnea on exertion, leg swelling, and weight gain and was found to have two histologically distinct cardiac masses: atrial myxoma with concurrent aortic fibroelastoma. Given her history of cirrhosis and end-stage renal disease, the patient was a poor surgical candidate but opted for excision of both masses. The patient eventually succumbed to her cirrhosis six weeks after presentation. In this report, we advocate for further research into medical management for the unique presentation of concurrent primary cardiac tumors in high-operative-risk patients, particularly those whose symptoms are mostly due to tamponade.

## Introduction

This was presented in the Medical Student/Resident Cardiothoracic Surgery Poster session at the virtual Chest Annual Meeting October 18-21, 2020. Atrial myxomas are the most common primary cardiac tumor, constituting half of all primary cardiac tumors and occurring predominantly in adults [1-3]. While the prevalence of primary cardiac tumors is approximately 3% overall, of those, 75% are benign and include other types such as lipomas, papillary fibroelastomas, and rhabdomyomas [3]. Cardiac tumors can be tricky to identify on initial presentation because symptoms will vary based on the tumor’s potential embolization, location, and size. Atrial myxomas tend to occur in middle age to late adulthood, much less so in the geriatric population [4]. While there have been reports documenting unique presentations of primary cardiac tumors, there is a paucity of literature surrounding conservative management in elderly, high-operative-risk patients with two concurrent primary cardiac tumors. We report a case of an elderly patient with two distinct histologically cardiac masses, initially presenting with symptoms of heart failure.

## Case presentation

**Figure 1:**
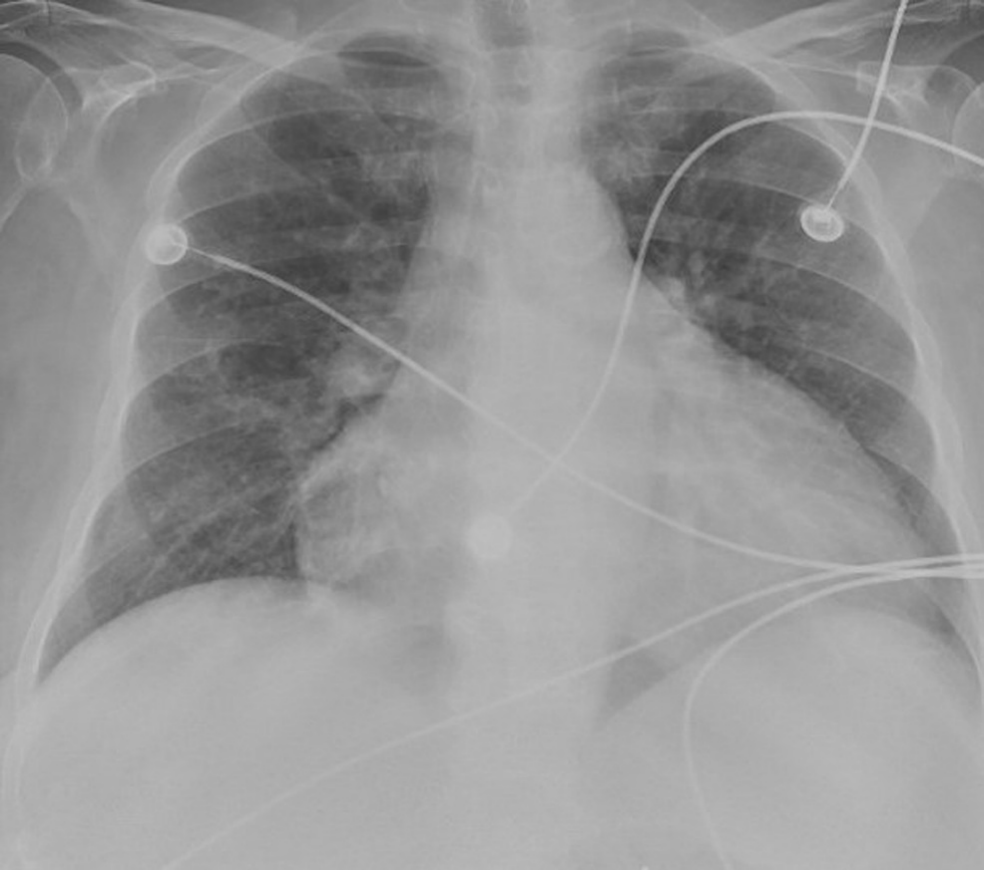
Chest x-ray (anterior-posterior view)

A 70-year-old female with a past medical history of squamous cell carcinoma, type II diabetes, rheumatoid arthritis, alcoholic cirrhosis with esophageal varices, tobacco abuse, and stage five chronic kidney disease presented to the emergency department with generalized abdominal pain, leg swelling, four weeks of dyspnea on exertion, and a weight gain of 29 pounds. She had been taking intermittent furosemide dosing at home with decreased response compared to baseline. Family history was notable for multiple types of cancer (ovarian, lung, breast), although the patient was up-to-date and negative for all relevant cancer screenings. She was hypertensive (154/58 mmHg), tachycardic (104 beats per minute), febrile (100.3 degrees Fahrenheit), and borderline tachypneic (20 breaths per minute) at presentation. Physical exam was notable for bilateral lower extremity 3+ pitting edema, an abdominal fluid wave, a systolic murmur at the left lower sternal border, and bibasilar crackles. Labs were significant for normocytic anemia, elevated blood urea nitrogen (BUN) and creatinine, hyponatremia, hypercalcemia, hypoalbuminemia, and elevated alkaline phosphatase. Troponins were negative, but the patient had elevated pro-B-type natriuretic peptide (proBNP). The chest radiograph demonstrated cardiomegaly (Figure 1).

**Figure 2:**
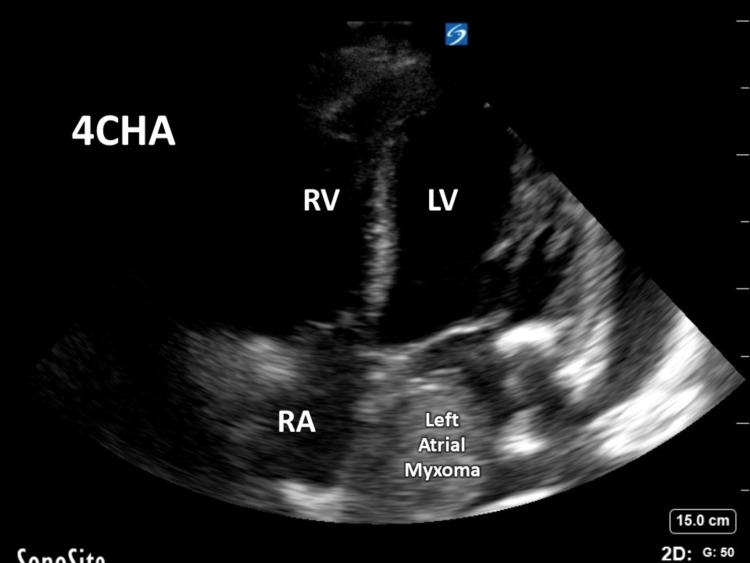
Transthoracic echocardiogram (TTE) left atrial myxoma 4-chamber view 4CHA - 4 chamber, LV - left ventricular, RV - right ventricular, RA - right atrium

**Figure 3:**
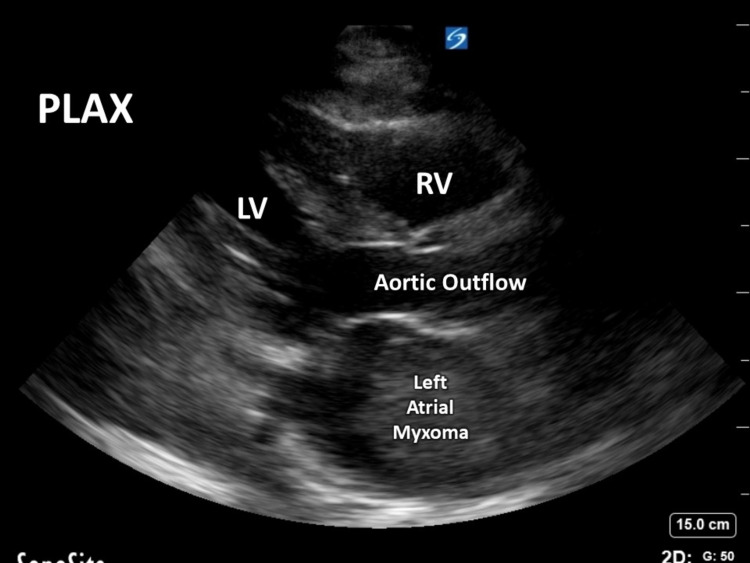
Transthoracic echocardiogram (TTE) left atrial myxoma parasternal long-axis view PLAX - parasternal long-axis view, RV - right ventricle, LV - left ventricle

A large pericardial effusion, abdominal ascites, and a left atrial mass that was echogenic were found with a point of care ultrasound. There were concerning signs for tamponade indicated by collapse of the right atria during diastole on the official 2D echocardiogram (Figure 2, 3).

**Figure 4:**
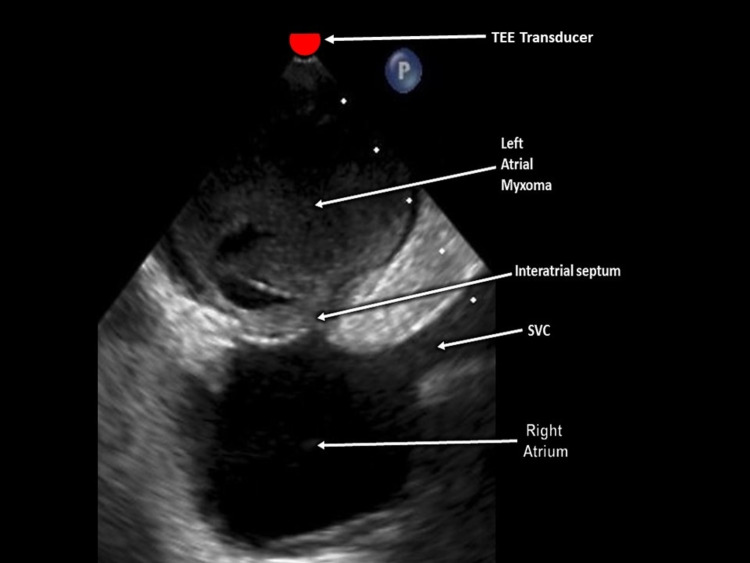
Transesophageal echocardiogram (TEE), left atrial myxoma SVC - superior vena cava

A total of 760cc of transudative fluid was drained via pericardial tap, resulting in the resolution of the patient’s symptoms. Two masses were evident on the transesophageal echocardiogram (TEE, Figure 4). The first was affixed to the septum between the atria, was well-circumscribed, and had some echolucency and measuring 3.8 by 4.5cm. The second mass was echogenic, measuring 4 by 5mm, and was located near the aortic side of the coronary cusp (left).

Conservative management was discussed, given her comorbid conditions. However, the patient chose to pursue surgery and, 10 days after presentation, cardiothoracic surgery excised the two masses. They reported a near annular mitral obstruction from a "ball valve" consequence of the mass. The pathology report of the specimens taken during surgery determined the masses to be respectively an atrial myxoma and an aortic fibroelastoma. The pathologist also commented that the pericardial biopsy showed evidence of sclerosing pericarditis that appeared chronic. Unfortunately, due to decompensated cirrhosis, the patient passed away after a prolonged hospital course and inpatient rehabilitation stay, approximately six weeks after presentation.

## Discussion

Cardiac tumors were once only something that could be studied during an autopsy, but in the last 64 years, the invention of 2-dimensional echocardiography, magnetic resonance imaging, and computed tomography have allowed physicians to diagnose such neoplasms prior to the death of a patient [5]. These cardiac tumors can be primary (benign or malignant) or secondary (always malignant) [5]. The occurrence of secondary cardiac tumors has been reported 100 times more common than primary tumors [5-7]. Assessment and treatment of cardiac tumors before significant symptoms leads to a better prognosis; however, the challenge for cardiologists, surgeons, and emergency physicians is the rarity of primary cardiac tumors, as well as a lack of knowledge of how to treat them for patients who are surgically high-risk [6,7].

The treatment of primary cardiac tumors is surgical [7]. The generally accepted treatment of these rare myxomas is operative resection [5]. Gupta et al. (2020) note that sufficient data to inform the creation of guidelines for cardiac tumors do not exist; thus, there is no guideline‐directed management of primary cardiac tumors for even a patient who is not as high-risk as our patient [7]. There are no known medicines that would shrink them or prevent them from growing further [5].

Our literature review resulted in only five documented cases of primary cardiac tumors that were both primary and different histologically; such cases are very uncommon [8-11]. Due to the untoward sequelae of mitral valve stenosis and embolization from the tumor, atrial myxomas and aortic fibroelastomas are generally treated with surgical resection [7]. However, for elderly populations with comorbid conditions, these interventions become far riskier. By undergoing surgical treatment, this patient risked exacerbating her kidney disease; as she was already at stage V, this would mean liver failure. Furthermore, it is likely that her acutely severe presentation was related to tamponade instead of stenosis of the mitral valve since pericardiocentesis relieved the patient’s symptoms. This indication potentially validates the option of medical management. Some studies have reported managing atrial myxoma conservatively for high-risk surgical patients [12, 13], yet no report exists on patients with concurrent cardiac tumors. Serial echocardiography to monitor and anticoagulation to prevent embolization could be suitable options for higher-risk patients, but because of the low disease prevalence, research on the long-term outcomes of this approach is limited [14, 15]. We advocate for a conservative approach in high-risk patients with concurrent primary cardiac tumors whose presenting symptoms are relieved by pericardiocentesis. Ultimately, further research on these types of patients is indicated.

## Conclusions

In this report, we detail a unique case presentation of atrial myxoma with concurrent fibroelastoma in an elderly female. There have been no prior studies detailing outcomes of non-surgical management in high-risk patients with concurrent cardiac tumors. In such patients presenting with correlative imaging findings whose symptoms are the result of tamponade rather than mitral valve obstruction, medical management could be a viable alternative, especially given prior studies demonstrating its efficacy in solo primary cardiac tumors.
